# Supplementary material for: Association analysis between an epigenetic alcohol risk score and blood pressure
Source: medRxiv. 2024 Mar 2:2024.02.29.24303545. Preprint. [Version 1] doi: 10.1101/2024.02.29.24303545 (PMC10925472; doi:10.1101/2024.02.29.24303545)
Supplement: Supplementary file 1 [file NIHPP2024.02.29.24303545v1-supplement-3.pdf]

medRxiv preprint doi: https://doi.org/10.1101/2024.02.29.24303545; this version posted March 4, 2024. The copyright holder for this preprint (which was not certified by peer review) is the author/funder, who has granted medRxiv a license to display the preprint in perpetuity.
It is made available under a CC-BY 4.0 International license .

Supplemental Figures

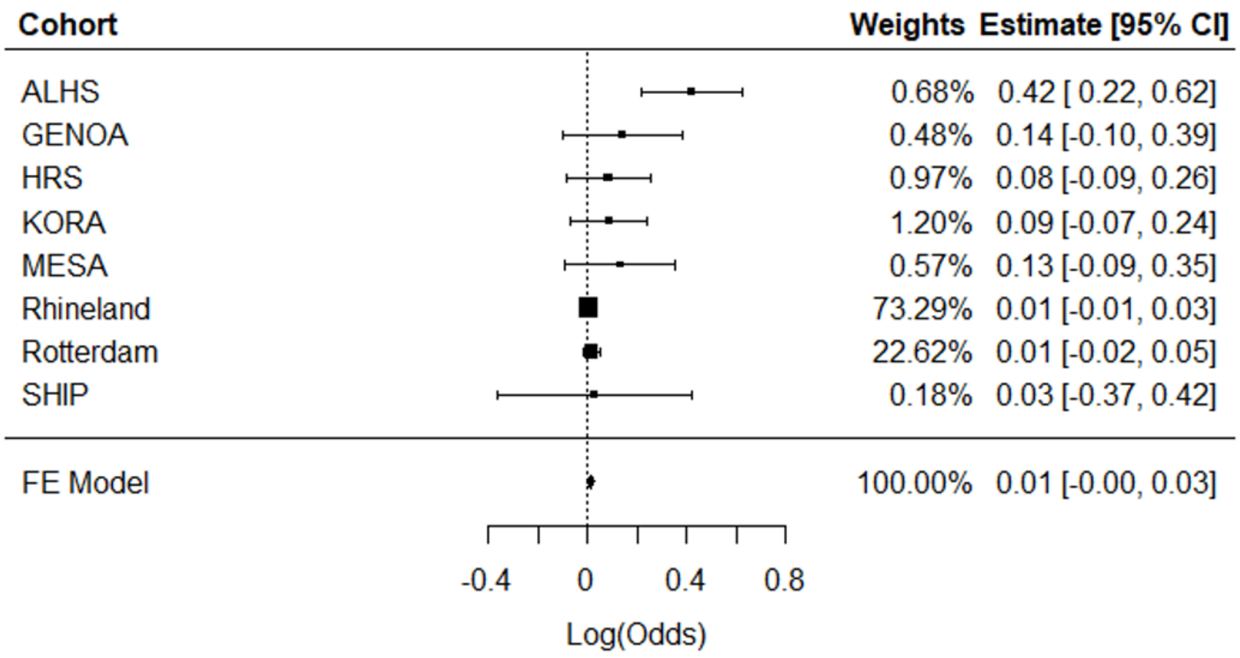

Supplemental Figure 1. Meta-analysis of cross-sectional association analyses of ERS in relation to hypertension in eight independent cohorts (n = 11,544). ALHS, Agricultural Lung Health Study; GENOA, Genetic Epidemiology Network of Arteriopathy; HRS, Health and Retirement Study; KORA, Cooperative Health Research in the Region Augsburg; MESA, Multi-Ethnic Study of Atherosclerosis; SHIP, Study of Health in Pomerania; FE, Fixed Effect; 95% CI, 95% Confidence Interval; log(Odds), log odds ratio.

medRxiv preprint doi: https://doi.org/10.1101/2024.02.29.24303545; this version posted March 4, 2024. The copyright holder for this preprint (which was not certified by peer review) is the author/funder, who has granted medRxiv a license to display the preprint in perpetuity.
It is made available under a CC-BY 4.0 International license .

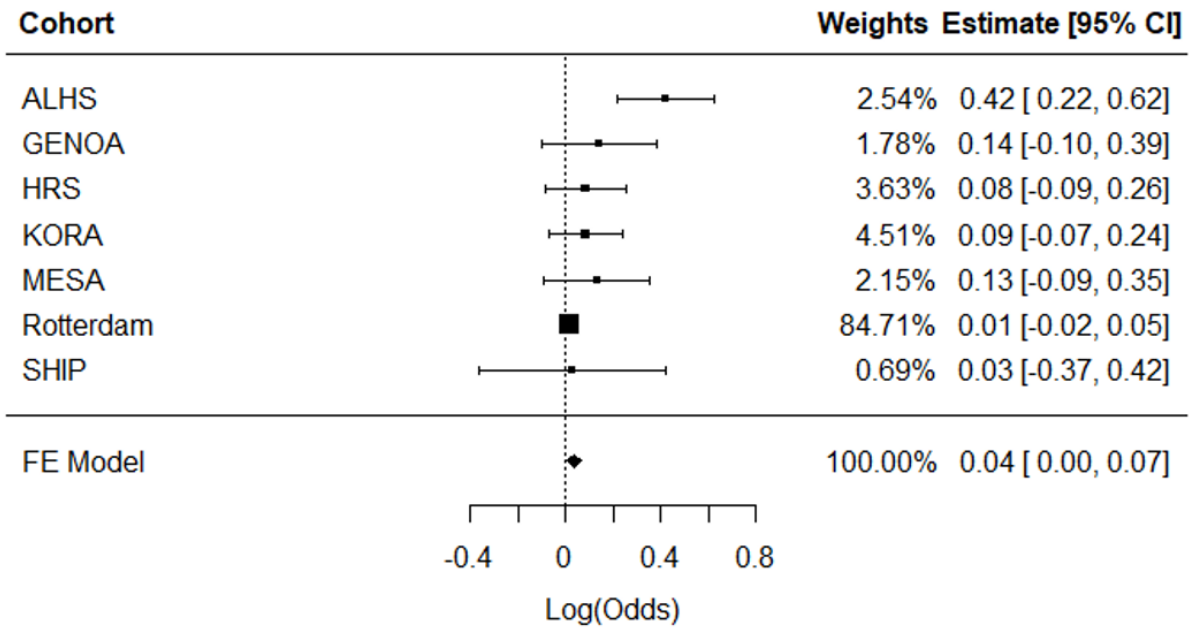

Supplemental Figure 2. Meta-analysis of cross-sectional association analyses of ERS in relation to HTN (excluding the Rhineland Study as sensitivity analysis). ALHS, Agricultural Lung Health Study; GENOA, Genetic Epidemiology Network of Arteriopathy; KORA, Cooperative Health Research in the Region Augsburg; HRS, Health and Retirement Study; MESA, Multi-Ethnic Study on Atherosclerosis; SHIP, Study of Health in Pomerania; FE, Fixed Effect; 95% CI, 95% Confidence Interval; log(Odds), log odds ratio.

medRxiv preprint doi: https://doi.org/10.1101/2024.02.29.24303545; this version posted March 4, 2024. The copyright holder for this preprint (which was not certified by peer review) is the author/funder, who has granted medRxiv a license to display the preprint in perpetuity.
It is made available under a CC-BY 4.0 International license .

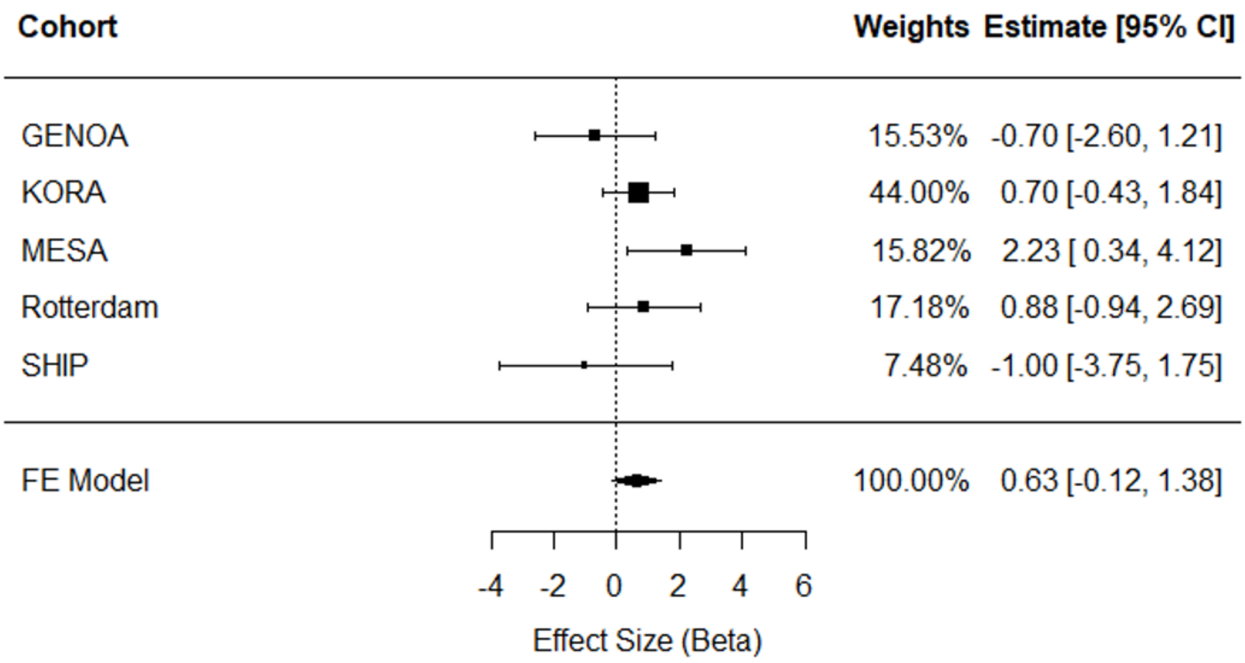

Supplemental Figure 3. Meta-analysis of longitudinal association analyses of ERS in relation to changes in systolic blood pressure in five independent cohorts (n=3,910). GENOA, Genetic Epidemiology Network of Arteriopathy; KORA, Cooperative Health Research in the Region Augsburg; MESA, Multi-Ethnic Study of Atherosclerosis; SHIP, Study of Health in Pomerania; FE, Fixed Effect; 95% CI, 95% Confidence Interval.

medRxiv preprint doi: https://doi.org/10.1101/2024.02.29.24303545; this version posted March 4, 2024. The copyright holder for this preprint (which was not certified by peer review) is the author/funder, who has granted medRxiv a license to display the preprint in perpetuity.
It is made available under a CC-BY 4.0 International license .

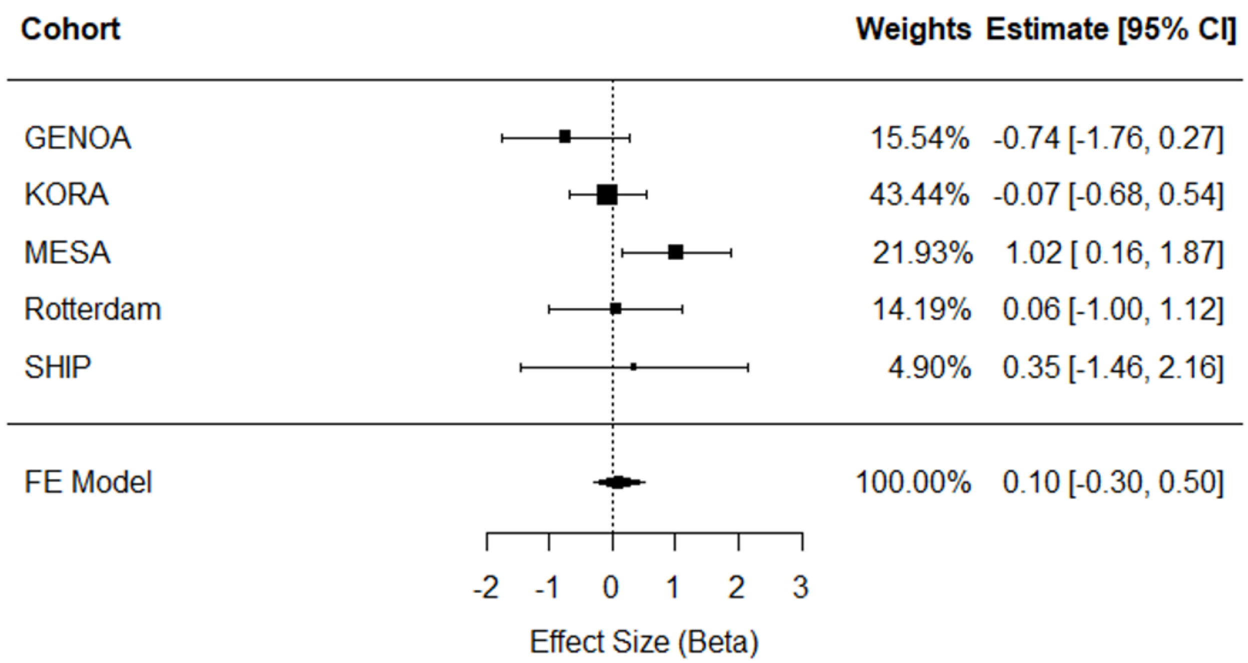

Supplemental Figure 4. Meta-analysis of longitudinal association analyses of ERS in relation to changes in diastolic blood pressure in five independent cohorts (n=3,910). GENOA, Genetic Epidemiology Network of Arteriopathy; KORA, Cooperative Health Research in the Region Augsburg; MESA, Multi-Ethnic Study of Atherosclerosis; SHIP, Study of Health in Pomerania; FE, Fixed Effect; 95% CI, 95% Confidence Interval.

medRxiv preprint doi: https://doi.org/10.1101/2024.02.29.24303545; this version posted March 4, 2024. The copyright holder for this preprint (which was not certified by peer review) is the author/funder, who has granted medRxiv a license to display the preprint in perpetuity.
It is made available under a CC-BY 4.0 International license .

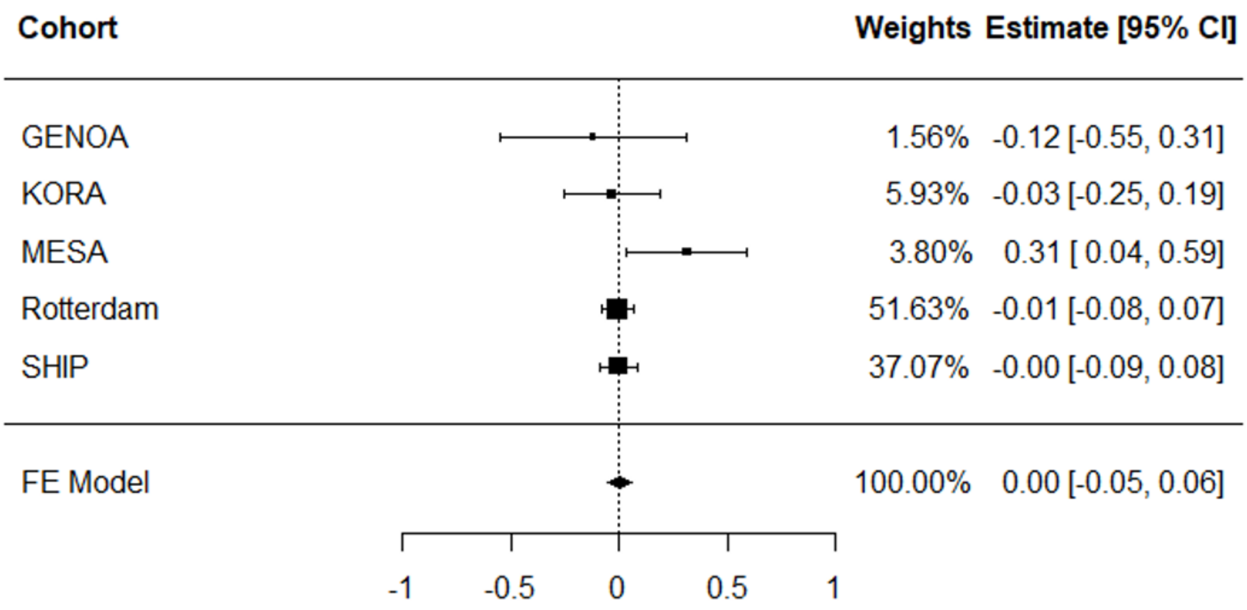

Supplemental Figure 5. Meta-analysis of ERS in relation to incident (i.e., new-onset) hypertension in five independent cohorts (n=3,228)**.** GENOA, Genetic Epidemiology Network of Arteriopathy; KORA, Cooperative Health Research in the Region Augsburg; MESA, Multi-Ethnic Study of Atherosclerosis; SHIP, Study of Health in Pomerania; FE, Fixed Effect; 95% CI, 95% Confidence Interval.

medRxiv preprint doi: https://doi.org/10.1101/2024.02.29.24303545; this version posted March 4, 2024. The copyright holder for this preprint (which was not certified by peer review) is the author/funder, who has granted medRxiv a license to display the preprint in perpetuity.
It is made available under a CC-BY 4.0 International license .

a. b.

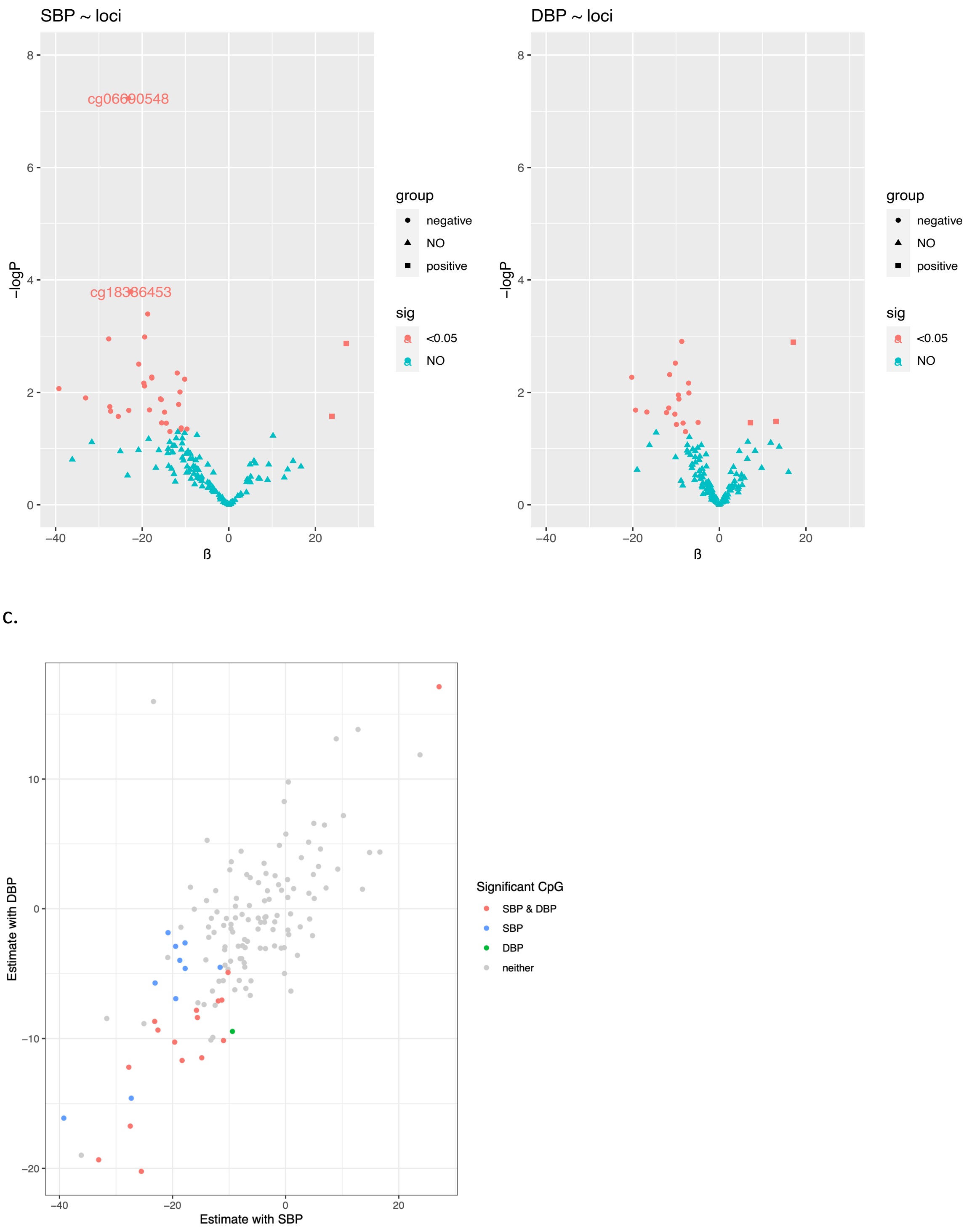

Supplemental Figure 6. Cross-sectional analysis of 144 CpG probes with blood pressure traits (i.e., SBP and DBP) in FHS. a) 144 CpG probes with SBP; b) 144 CpG probes with DBP; c) comparison on estimates and p-value of 144 CpG probes between SBP and DBP. FHS, Framingham Heart Study; SBP, systolic blood pressure; DBP, diastolic blood pressure.
